# Adolescents’ Experiences of Idiopathic Scoliosis in the Presurgical Period: A Qualitative Study

**DOI:** 10.1093/jpepsy/jsab095

**Published:** 2021-09-15

**Authors:** Gillian S Motyer, Patrick J Kiely, Amanda Fitzgerald

**Affiliations:** School of Psychology, University College Dublin and; Department of Orthopaedics, Children’s Health Ireland at Crumlin, Ireland; School of Psychology, University College Dublin and

**Keywords:** adolescents, chronic illness, psychosocial functioning, quality of life

## Abstract

**Objective:**

Adolescent idiopathic scoliosis (AIS) is a sideways curvature of the spine that can progress severely during adolescent development and require surgical intervention. This qualitative study was conducted to explore the psychosocial experiences of adolescents with idiopathic scoliosis during the presurgical stage of treatment.

**Methods:**

Fourteen adolescents with moderate-to-severe AIS aged 12–17 years participated in semistructured interviews and data were analyzed using inductive reflexive thematic analysis.

**Results:**

Four key themes were generated from the analysis. “Proceeding with Caution” described adolescents’ adaptation to the physical impact of their AIS, while “Am I Different?” encompassed adolescents’ perceptions of their changing appearance and visibility of their condition. “An Emotional Journey” captured the rollercoaster of emotions from shock at diagnosis to the daunting realization of the severity of their condition, while knowing others with AIS could ease the emotional burden. Finally, adolescents’ concerns and expectations about their prospective surgery were captured by the theme “No Pain, No Gain”, whereby they were often keen to put surgery behind them.

**Conclusions:**

Understanding and addressing adolescents’ psychosocial support needs as they manage the challenges associated with idiopathic scoliosis is a key component of promoting better outcomes among this patient group. Clinical implications and opportunities for support provision are discussed.

## Introduction

Adolescent idiopathic scoliosis (AIS) is a sideways curvature of the spine that is typically diagnosed in the early teenage years (Weinstein et al., 2008) and is one of the most common pediatric spinal conditions seen by primary care physicians, pediatricians, and orthopedic surgeons, affecting approximately 1–3% of adolescents (Altaf et al., 2013). Although the etiology of AIS is uncertain, growth spurts during adolescent development are understood to contribute to progression of the spinal curve (Beauchamp et al., 2019). Curve progression can be associated with symptoms including postural asymmetries, imbalanced shoulders and hips, rib prominence, back pain, and pulmonary issues (Weinstein et al., 2008; Altaf et al., 2013). Living with this often progressive condition can be burdensome for adolescents, who are already experiencing the normative developmental challenges of adolescence including pubertal changes, increasing independence from parents, and a desire for peer conformity. Adolescents with AIS are more likely to report appearance self-consciousness, associated problems with social interactions (Auerbach et al., 2014), and higher levels of body image dissatisfaction compared with nonaffected peers (Tones et al., 2006; Rushton & Grevitt, 2013). Considering that AIS can be associated with body image disturbances, concerns have also been raised that adolescents with AIS may be vulnerable to other psychosocial difficulties such as symptoms of depression or anxiety (Gallant et al., 2018). Research has indicated that patients with scoliosis are almost twice as likely to develop depression in comparison to nonaffected individuals (Chang et al., 2016), and that those living with AIS experience similar levels of anxiety symptoms to pediatric patients with cancer (Sanders et al., 2018).

Treatment options for AIS include conservative orthotic bracing and specialized physiotherapy programs which aim to reduce curve progression (Beauchamp et al., 2019). However, about 10% of those with AIS develop severe curvatures that require surgical intervention (Altaf et al., 2013). Posterior spinal fusion is the common surgical approach in AIS which involves insertion of metal rods and screws to hold the spine in place, with modern techniques having relatively low rates of complications (Altaf et al., 2013; Beauchamp et al., 2019). For adolescents whose spinal curvatures exceed a Cobb angle of 45–50°, surgery is generally recommended with the aim of preventing future progression, correcting the existing curvature, and improving quality of life (Weinstein et al., 2008). The period of presurgical preparation is a challenging time for adolescents with AIS, who are living with significantly progressing spinal curvatures and anticipatory stress in advance of their prospective surgery (Rullander et al., 2016).

Despite the significant symptoms associated with AIS and the seriousness of undergoing spinal fusion surgery, limited research has sought to explore adolescents’ psychosocial experiences specifically during the presurgical stage of treatment. Studies by Chan et al. (2017) and Bridwell et al. (2000) surveyed patients with AIS prior to surgery and identified primary surgical concerns as ‘pain’, ‘ability to return to activities after surgery’, and the possibility of ‘surgical complications’ (e.g. neurologic injury). More recently, Lonner et al. (2020) surveyed 44 patients about the most important aspects of their lives that were affected by AIS in the presurgical period and found that ‘sports’, ‘general function’, and ‘general fitness’ were the most common concerns, and that improving their ‘self-esteem’ and ‘pain’ were the most common operative aspirations.

MacCulloch et al. (2009) qualitatively investigated information and support needs among 11 adolescents with AIS who had undergone or were anticipating spinal surgery. Participants endorsed development of a customized website including information on topics including what to expect at the hospital, managing recovery at home, and how school or peer relationships may be impacted postsurgically. To meet support needs, adolescents wanted the resource to include the opportunity for peer support and interactive discussion where personal stories could be shared. While MacCulloch et al. highlighted important considerations for informed surgical decision-making, the focus on surgical information needs precluded a more holistic exploration of adolescents’ experiences throughout the presurgical period. Other qualitative studies on AIS have described challenges associated with brace wear (Donnelly et al., 2004) or focused on the postsurgical period, exploring adolescents’ experience of undergoing surgery, postoperative pain, and recovery (Rullander et al., 2013; Bray & Craske, 2015; Perry, 2018).

This study will address the need for a more comprehensive and in-depth exploration of the psychosocial experiences among adolescents living with presurgical idiopathic scoliosis. Adolescents at the presurgical stage of AIS treatment are living with significantly progressed spinal curvatures that have potential to negatively impact their psychosocial functioning and wellbeing. The presurgical period is however an opportune time to provide psychosocial support, as adolescents are typically reviewed clinically every 4–6 months to monitor curve progression during growth (Altaf et al., 2013) and are linked in with a multidisciplinary team. Understanding the experiences and perspectives of adolescents throughout the presurgical period is a key step in informing patient-centered care throughout this challenging stage of treatment. Such insight would allow identification of possible support needs and how they could be addressed, information provision that is tailored to adolescents’ concerns, and improvement of preoperative preparation for this patient group. The aim of this study was therefore to explore adolescents’ experiences of living with presurgical idiopathic scoliosis, with a focus on their psychological and social functioning and wellbeing.

## Methods

This study adopted a qualitative approach in order to explore the experiences of presurgical adolescents with AIS. Qualitative methods are recognized as a valuable approach in gaining an in-depth understanding of the perspectives and needs of pediatric patient groups (Berlin et al., 2017, p. 47) and this approach has thus far been underutilized in AIS research.

### Participants and Recruitment Procedures

Fourteen adolescents with AIS were recruited to participate via the orthopedic department of an Irish children’s hospital, a national tertiary referral center for pediatric scoliosis care. Adolescents were eligible to participate if they were aged 12–18 years, diagnosed with AIS, and were presurgical candidates who had not yet undergone surgical intervention for AIS. A purposive sampling technique was employed to recruit both males and females meeting the inclusion criteria from urban and rural locations across Ireland. Between October 2018 and May 2019, eligible adolescents and their parent or guardian(s) attending a routine presurgical outpatient appointment for their scoliosis were informed about the study by a spinal nurse specialist to assess their interest in participation. The purpose of the study, to understand the experiences of adolescents with presurgical AIS, was explained to potential participants. During recruitment, the parents of 16 adolescents agreed to be contacted by the research team with further information and, of these, 14 chose to participate. Reasons for nonparticipation included undergoing surgery before an interview date could be scheduled and unsuitable timing of the study. The first author followed up with each parent by phone or email and arranged a meeting for data collection either in the hospital or participant home setting. Participants and their parents were provided with detailed age-appropriate study information materials (printed and/or via email) prior to data collection and were given the opportunity to discuss any queries. Parents provided written consent and adolescents provided written assent to participate.

The final sample included 10 females and 4 males (*N *=* *14) with moderate-to-severe thoracic or thoracolumbar scoliosis, aged 12–17 years (*M *=* *14.6). The higher proportion of females in the sample was reflective of the higher incidence of AIS among females (Konieczny et al., 2013). All participants identified as White Irish. Mean time since diagnosis was 12.7 months (range: 3–26 months). At time of interview, all participants were considered surgical candidates based on the size of their spinal curve and/or potential for future curve progression and had discussed prospective surgery with their medical team. Cobb angles of the major scoliotic curves on participants' most recent radiographs ranged from 46° to 100° (*M *=* *68°, *SD* = 14.8). At the time of the study, two participants had a confirmed surgery date. The remainder were waiting to receive a date or anticipated that they would be scheduled for surgery in future. Two participants had also worn an orthotic brace as part of their treatment. Participant characteristics are summarized in Table I.

**Table I.** Participant Characteristics (*N* = 14)

Variable	Range	*M*
Age (years)	12–17	14.6
Curve size^a^	46–100°	68°
	*n*	%
Gender		
Male	4	28.6
Female	10	71.4
Race and ethnicity		
White Irish	14	100
Home setting^b^		
Urban	5	35.7
Rural	9	64.3
Length of diagnosis		
<1 year	5	35.7
1–2 years	8	57.1
2–3 years	1	7.1
Curve type		
Thoracic	6	42.9
Thoracolumbar	8	57.1
Treatment details		
Bracing	2	14.3
Awaiting surgery	10	71.4
Surgery scheduled	2	14.3

a Curve size is the surgeon assessed Cobb angle measurement of the major scoliotic curve.

b Based on the Central Statistics Office geographical classification guidelines.

### Data Collection

Prior study approval was obtained from the relevant research ethics committees at the hospital and university. The first author, a PhD candidate in Psychology, conducted all of the interviews in person, either in the participant’s home (*n *=* *11) or in the outpatient department of the hospital (*n *=* *3). Before interview, a brief demographic questionnaire was used to collect detail on participants’ age, gender, ethnicity, home setting, length of diagnosis, and treatment status. The semistructured interview schedule, developed based on prior literature, consisted of open-ended questions and related probes that encouraged participants to discuss their experiences of living with AIS. Interview topics covered the diagnosis of scoliosis, an exploration of what areas of their lives were affected by scoliosis, concerns, or challenges associated with scoliosis, body image, and their medical treatment (i.e. prospective surgery). A summary of the main interview questions is presented in Table II. Interviews were audio-recorded and lasted between 15 and 48 min (*M *=* *32). Participant recruitment and data collection ceased when authors G.S.M. and A.F. judged that the dataset contained sufficient information power to generate meaningful themes and no new topics of discussion were being introduced in interviews. Information power relates to whether the data demonstrate the required breadth and depth relative to factors including the homogeneity of the sample and the scope of the study (Morse, 2000; Malterud et al., 2016) and is a helpful concept in determining appropriate sample size in exploratory inductive qualitative studies. Interview data were transcribed verbatim, and any potentially identifying information was anonymized at the stage of transcription.

**Table II.** Summary of the Interview Schedule

Can you tell me about when you first found out that you had scoliosis? Could you tell me about the treatment you have been receiving for your scoliosis? Is there anything in your life that you think is affected by your scoliosis? Do you have any concerns about your scoliosis? Have your thoughts and feelings been affected by your scoliosis? Some people with scoliosis can experience changes to their body, do you think your scoliosis affects the way that you look? Can you tell me about how you cope with having scoliosis? Are there any resources or services that have been helpful in supporting you?

### Data Analysis

Data were analyzed using inductive reflexive thematic analysis (TA; Braun & Clarke, 2006, 2019). Reflexive TA is a method that involves a process of systematically coding patterns of meaning across the dataset to develop a detailed and rich account of participant experiences through analytic narrative and data extracts. This analysis is particularly suited to analyzing interview data and the inductive approach suited the exploratory nature of our research as we focused on developing themes based on the data rather than utilizing predetermined codes. Following the steps of reflexive TA, the first author conducted initial coding of the transcripts with an inductive approach (i.e. exploratory and data-driven). Coding was an iterative process that involved revisiting transcripts and reviewing codes as the analysis progressed. G.S.M. and A.F. independently coded approximately one-third of the dataset at the beginning of the coding phase and again midway through analyses. They met regularly to collaboratively discuss interpretations of the data and theme development to promote a rich and rigorous analysis. Finally, codes were clustered into broader units of meaning which were refined to create themes and a narrative describing each theme was developed. NVivo 12 software was used to facilitate data management.

Following guidance on establishing rigor in qualitative research (O’Brien et al., 2014; Nowell et al., 2017), strategies to ensure credibility of the findings were incorporated. In line with reflexive TA (Braun & Clarke, 2019), critical self-evaluation of the researcher’s influence (e.g. reflective notes, debriefing) alongside the collaborative approach to data analysis (described above) facilitated engagement in researcher reflexivity and promoted a thorough analysis that was grounded in the data. A clear account of participant recruitment and the characteristics of the sample were provided to contextualize the findings and provide guidance on transferability. Quotations representative of participants’ experiences across the dataset were embedded throughout the thematic narratives, and participants are specified by participant numbers (e.g. P1) followed by M or F (male or female), and their age.

## Results

Analysis of the interview data generated four key themes which reflected pertinent aspects of adolescents’ lived experiences prior to surgery. Themes included “Proceeding with Caution,” “Am I Different?” “An Emotional Journey,” and “No Pain, No Gain.” Thematic findings and illustrative data extracts are presented in Table III.

**Table III.** Thematic Findings

Themes	Sample data extracts
1. Proceeding with caution	“I used to be able to play hurling in P.E. and I can’t do that anymore now because we’d be kind of rough with it.” (M, 14). “I’m just making sure I’m not straining it too much just in case it might get worse.” (F, 16).
2. Am I different?	
2a. Appearance changes	“it’s twisted my ribs and I have a lump here [on my side].” (M, 16). “I was just like looking at myself in the mirror and I was like my body looks a little bit odd, like it kind of looks lopsided…” (F, 15).
2b. A hidden condition	“I asked a few of my friends [about my AIS] and some of them said that they actually never realised.” (F, 15).
3. An emotional journey	
3a. Emotional rollercoaster	“I was just a bit shocked [at diagnosis] 'cause like I didn’t know what it was and I didn’t know if it was real threatening or anything.” (F, 16). “I think a lot about my back and kinda how scary the operation might be and sometimes I get into a panic and my back starts to hurt even more.” (F, 16).
3b. Not the only one	“It’s just knowing that there’s so many other people as well it’s reassuring that you’re not kind of… different I suppose.” (F, 15).
4. No pain, no gain	“The surgery will make me less conscious of my back, and I can wear a normal top again instead of the big baggy ones.” (F, 14). “I think the surgery kind of scares me a bit but I know it’s going to help me and, in the end, I know it’s the right thing to do.” (F, 13).

*Note*. F, female; M, male, followed by age.

### Theme 1: Proceeding with Caution

Participants approached activities with increased caution as they managed the physical symptoms of their scoliosis and avoided straining their back in the presurgical period.

It was evident that adolescents were cautious of activities that may aggravate their condition. Pain or discomfort was commonly discussed and could occur when sitting for long periods at school, when carrying bags, or after physical activity. Some of the adolescents appeared to be aware of their own limits, and avoided activities that could cause back pain and they rested when needed. This was demonstrated by an adolescent who recalled attending a school trip where she could not participate to the same level as her peers: “they were doing emm, kind of like bungee jumping and I just chose not to do it because I went rock climbing with them before that and I was in bits after so I just didn’t want to risk it … it looked fun though.” (P5; Female, 16). Despite avoidance of certain activities or sports, it was evident that most of the adolescents continued to remain active, albeit with increased caution; “I do taekwondo and I can’t do like kind of self-defence where you knock people to the ground anymore because obviously my back would get hurt.” (P4; Female; 14).

Some adolescents could no longer participate in sports and activities to the same level as before their scoliosis, which could lead to feelings that they were missing out. This was particularly evident for high impact or contact sports; “…my friends started after me doing boxing, and then they got to go on, and like, go to all the competitions and win all the medals and stuff like that, and I found out [that I had scoliosis] just before I was going for the competition level, and I couldn’t do it.” (P9; Male, 14). As well as avoiding specific activities, day to day life could take its’ toll physically; “you have to be careful, and you know I might say well I’m only going to go out for that long because I’ll have to go lie down then for a while and just take it easy.” (P2, Female, 13).

Adolescents felt that it was important to follow advice from medical staff in relation to what to avoid, what activities could be helpful, as well as keeping healthy in advance of their surgery; “I just try and do everything the doctors say like eat healthy, like vitamins… and the dietician was like you need to be like, perfect health and stuff, so listening to them and going swimming and everything, that like helps me feel better cause like how could it go wrong then.” (P7; Female, 15). This participant, like others, had taken up swimming since their diagnosis, a form of exercise often recommended for those with AIS as a noncontact activity that can increase fitness and mobility in advance of undergoing surgery.

Importantly, for adolescents who were participating in sports and exercise, they felt it was helpful to them in the presurgical period; “Just the fact that I play sport kinda helps me like and just gets my mind off [my scoliosis], I would just totally forget about it there.” (P3; Female, 12). As noted here, at a time when preferred activities may be on hold, taking part in exercise as recommended by the medical team may be just as beneficial for adolescents’ mental health as it is for maintaining presurgical fitness.

### Theme 2: Am I Different?

The second theme describes the experiences of adolescents as they process their changing bodies and question how their appearance may be perceived by others, including two subthemes. *Appearance Changes* focuses on adolescents’ perceptions of their condition and how it has affected their appearance. Relatedly, *A Hidden Condition* captures how adolescents manage the visibility of their condition and how they may navigate this in the context of their peers.

#### Appearance Changes

All of the adolescents acknowledged changes in their physical appearance as a result of their scoliosis. Changes were typically associated with truncal asymmetries, as adolescents described their backs, shoulders, ribs, hips, and posture as uneven. Noticing irregularities in their appearance was often what led adolescents to seek medical advice and subsequently be diagnosed with AIS; “When I was getting out of the shower one day I just kinda noticed in the mirror like, it was kinda odd like, a bump at the lower back and my shoulder blade was kind of gone out a bit […] I had no idea what it was, I thought maybe it could be a tumour or something.” (P10; Male, 17). Similar to this adolescent, many could remember the first time they noticed visible signs of their spinal condition and the confusion that surrounded it.

Throughout interviews, words were used which indicated that participants perceived their condition as “abnormal,” and they felt different to their peers; “When we were all getting in our swimsuits and going to the beach and all that… I didn’t want to get into my swimsuits because my body didn’t look right.” (P6; Female, 15). For some participants, the impact that their scoliosis had on their appearance was particularly burdensome, as articulated by an adolescent who felt she could no longer wear clothing that she liked and that corrective surgery was necessary in order for her to be happy with her body again: “I wouldn’t be able to live with it… because I just wouldn’t be able to like my body.” (P7, Female, 15).

There was variation among adolescents in relation to appearance concerns, as half of the participants indicated that in general, they were content with their looks despite scoliosis-related appearance changes. It was apparent in some instances that there may have been a disconnect between how adolescents’ felt about the appearance of their scoliosis and how they felt about their overall or general body appearance. For example, this was demonstrated by an adolescent who felt she was “very different looking” from her peers and was concerned about how she looked in certain clothes, but also mentioned that she was a “confident person,” and that she was reasonably happy with her looks: “like it does bother me that I have it [scoliosis] but emm, I don’t mind looking the way I do.” (P5, Female, 16). Another adolescent described a number of visual symptoms of her scoliosis (e.g. unlevel shoulders) but also perceived that her body image satisfaction was similar to other teenage girls: “They [appearance changes] don’t really bother me too much, like I wouldn’t be 100% happy with how I look, but I think that’s even what most girls are like really.” (P13, Female, 16).

#### A Hidden Condition

Although adolescents observed changes in their appearance, there was uncertainty as to whether others could see the physical changes in their bodies, or how visible their scoliosis was to others. Many adolescents believed their scoliosis was not very noticeable; “I don’t think they (peers) notice it, being honest, I never talk to them about it. I think it’s just me because I see it every day.” (P11, Female, 14). They acknowledged that their scoliosis would be more visible in certain situations such as when swimming, wearing tighter or more revealing clothing, or while getting changed for P.E. at school.

Some adolescents made efforts to disguise or hide their condition from others: “when I found out I was very insecure I didn’t know what it looked like to other people, and I was trying to like, straighten it as much as I could…” (P8, Female, 15). Different camouflage strategies discussed by adolescents included wearing bigger or “baggy” clothing, being vigilant of their posture, and changing quickly at school. One adolescent reported actively trying to reduce her body asymmetry by losing weight and building muscle at the gym.

The perception of scoliosis as “abnormal” and a condition of limited awareness within the population seemed to exacerbate adolescents’ concerns about others seeing their scoliosis; “If someone just broke an ankle or broke a bone you know you wouldn’t really second look it, I feel like [scoliosis] is definitely something that people would look at and look again at, and be kind of like, ‘oh what’s that?’ and it’s kind of different, it’s a lot different to what friends or you know, what they would look like.” (P2; Female, 13).

Some adolescents demonstrated a reluctance to talk with their peers about scoliosis, either because they felt they were unable to explain it or because speaking about it would make them uncomfortable. For example, a participant had asked a peer to be his date for an upcoming social event, but worried about the fact that he had not told her about his scoliosis and potential surgery: “Honestly I’d feel a bit relieved [if I told her], but honestly I just don’t know how to say it like in the right way.” (P10, Male, 17). Another adolescent had started to avoid going to youth club as he felt his scoliosis was becoming more noticeable and did not want to have to explain it anyone: “I was trying to get it off my mind, stop thinking about it the whole time, and I’m like, if people were bringing it up the whole time I didn’t want that happening so […] I just didn’t want the hassle of explaining it to them to be honest.” (P9, Male, 14). There appeared to be a desire to maintain normality for many adolescents as they did not want to dwell on their scoliosis and endeavored to “get on with it.”

Telling friends about their scoliosis came across as a difficult step, and while some adolescents appeared to tell close friends as a way of seeking social support, others may have told their friends out of necessity. For example, explaining why they missed school for hospital appointments or why they were not taking part in particular sports anymore. For those who told friends about their scoliosis, some peers had initially been surprised at the diagnosis indicating that the adolescents’ scoliosis may not have been noticeable at the time. Confiding in close friends appeared to be helpful to adolescents, as they felt their friends reacted well and were supportive: “it’s nice to be able like… them not judging you about it like it’s nice to be able to talk to them about how sometimes it’s painful or. sometimes you can’t do things, like that you don’t want to mention to other people.” (P14, Female, 12).

### Theme 3: An Emotional Journey

From the time of their scoliosis diagnosis to preparing for surgery, it was evident adolescents could experience an Emotional Rollercoaster as described by the first subtheme. However, knowing that they were not alone in their experience of AIS could provide adolescents with support along this emotional journey as captured by the second subtheme *Not the Only One.*

#### Emotional Rollercoaster

Many adolescents recalled their shock and distress around the time of their diagnosis; “I was definitely, my head was spinning a bit, I was thinking about everything that could happen and, just because I wasn’t really expecting it to look like that at all, especially seeing the S [curvature on the X-ray].” (P13, Female, 16). The majority of adolescents recalled having limited or no previous knowledge of scoliosis. For the couple of adolescents who had some previous exposure to the condition, their prior knowledge of the condition contributed to fear at diagnosis, perhaps because they were aware of the potential severity of the condition: “I remember like seeing it on the Late-Late show with that girl and I said to mam, aww that’d be like the worst thing ever to have, and then like a week later I found out I had it so I was really upset.” (P11, Female, 14).

While some adolescents were clearly emotionally distressed and apprehensive following diagnosis of their condition, it appeared that others went through a process of change in their emotions, as they realized the severity of their condition over time. Reaching the stage of surgical consideration could be interpreted as a turning point for many adolescents, who may have not realized the severity of their condition until surgery was suggested as a treatment option by their medical team; “Well I… when the word was first thrown out I didn’t have any idea what scoliosis was emm, but it was explained to me what it was, and, well I wasn’t really that worried at the time, but, I think, now like, recently, like it’s kinda dawned on me.” (P10, Male, 17).

Adolescents could feel particularly down when their scoliosis caused them to miss out on activities with friends or impeded their capabilities in sports and activities. The process of choosing clothes to wear was also challenging for some as this could highlight appearance-related issues. The prospect of undergoing spinal surgery understandably caused fear and anxiety, which was likely to be heightened as the surgery date neared. For example, an adolescent whose surgery was scheduled for less than 2 weeks following the interview described how the thought of surgery could influence her mood: “I was at my friend’s house a while ago and we were all having great fun and all that and then it [surgery] came into my head… and I actually wouldn’t talk to anyone for about 20 to 30 minutes because I started freaking myself out you know?” (P6, Female, 15). Despite their fears, adolescents also demonstrated considerable optimism in relation to their prospective surgery as they looked forward to improvements in their condition postoperatively.

#### Not the Only One

While experiencing a rollercoaster of emotions throughout the presurgical period, adolescents could gain some reassurance through realizing they were not alone. In coming to terms with their condition, many adolescents gained emotional support through meeting or hearing stories about other young people with scoliosis, and through this they felt less isolated and could relate to the challenges that each other were going through. This was reflected in a participant’s experience at a swimming group for young people with scoliosis: “when you’re there you kind of feel like there’s other people and there’s always someone worse than you and you feel… it’s going to be okay like I’m not the only one.” (P2, Female, 13). Some adolescents thought it was particularly helpful to meet someone who had already been through surgery as they gained practical advice about what to expect at the hospital and during recovery. Seeing the results of surgery provided reassurance; “I was actually talking to one girl and I think she got [surgery] when she was like seventeen and it makes you more relaxed when you talk to people, like knowing that you’re actually going to be fine after surgery.” (P5, Female, 16).

### Theme 4: No Pain, No Gain

This final theme centered on anticipation of post-surgical benefits despite fears or concerns about surgery. Although the prospect of undergoing major surgery was a stressor for many adolescents, the operation also represented a life-changing event with positive outcomes and an opportunity to regain normality. This balance was reflected in adolescents’ beliefs about their upcoming surgery; “I’m nervous to get it done, but I know I’ll be glad when it’s over, ‘cause in the long term as I said it would just be really good and helpful, and make me feel good, better about myself as well.” (P13, Female, 16).

Surgical concerns related to possible complications and the subsequent recovery period. Concerns included the risk of paralysis (although also acknowledged as unlikely), the appearance of the surgical scar, and the possibility of damaging their spinal fusion postoperatively. A couple of adolescents doubted the permanency of the spinal fusion by questioning if their scoliosis could return in future. Concern about their lives being interrupted by the surgery was also evident, as they anticipated a challenging recovery and considered what they might fall behind on in school, sport, or social activities. Adolescents were often keen to put the surgery behind them so that they could get on with their lives; “honestly I think I just want it to be done and over with. It’s a case of going in, it’s over and then the recovery.” (P6, Female, 15).

Long-term, many adolescents felt that undergoing surgery was important to prevent their scoliosis from progressing further and to avoid a more debilitating curvature in the future. Many also anticipated an improvement in the appearance of their body following surgery. Other positive outcomes included regaining normality in their lives, the ability to play various sports, and improved self-esteem. It was apparent that for some adolescents having surgery was believed to be the solution to the problems they were experiencing; “Once I get the surgery done I feel like I’ll be able to get back to normal.” (P4, Female, 14).

Similarly to how the pros and cons of surgery were acknowledged, the two adolescents who wore a torso brace as part of their treatment reflected on both negative and positive aspects of their brace. Despite challenges including discomfort and a reluctance to wear the orthosis in social settings, adolescents appreciated the prognostic benefits of their brace; “Well I don’t mind wearing it, like I know it’s doing good for me so that’s always like a kind of lift up because I know it’s doing me better.” (P14; Female, 12). These adolescents looked forward to no longer wearing the brace postoperatively.

Gaining reassurance from the benefits they anticipated postoperatively allowed adolescents to remain hopeful and focused on the future, which appeared to give less weight to the challenges they were experiencing in the presurgical period. This was articulated by an adolescent in relation to appearance: “Once I get the surgery the whole look of it going to change. Right now, I’m not a huge fan of the way it looks but you know you just kind of have to, that’s just the way it is, so em, I am unhappy about that but em I know that will change so kind of have that comfort I suppose.” (P2, Female, 13). Adopting an optimistic mindset and focusing on an ideal surgical outcome in this way appeared to facilitate coping throughout the presurgical period.

## Discussion

This study explored the experiences of presurgical adolescents with AIS from a psychosocial perspective. Four key themes with associated subthemes were developed which provided new insights into the experiences of this patient group and considerations for their clinical care and psychosocial support.

Adolescents’ physical limitations and caution to avoid pain or injury are common experiences among young people with physical health conditions, with similar reports of “missing out” found in research concerning juvenile idiopathic arthritis (Tong et al., 2012). Despite physical constraints, many adolescents in this study continued to participate in sport and exercise. This is positive given the benefits of physical activity for overall health, aerobic capacity, and wellbeing in patients with scoliosis (Kakar et al., 2017), and also considering the importance of recreational activities for social skills and friendship development during adolescence. However, adolescents with AIS may be at risk of reduced participation in physical activity due to factors such as back pain and fear of injury (Kakar et al., 2017). It is generally recommended that those with AIS remain active where possible, however there is no definitive guidance available on suitable levels or types of physical activity for AIS at various stages of treatment (Tarrant et al., 2014). Adolescents were keen to follow guidance from their medical team at the presurgical stage and can seek clarity on activity participation in the clinical setting. The medical team therefore plays an important role in using their expertise to guide those with AIS on activity participation throughout treatment.

Adolescence is a time of heightened appearance-related pressure (Rumsey & Harcourt, 2012) and developing an appearance altering condition such as scoliosis can threaten a desire for conformity with peers and healthy body image development (Auerbach et al., 2014; Gallant et al., 2018). Our findings demonstrated that although some adolescents were unhappy with the appearance of their scoliosis, others indicated they were generally happy with their appearance. Possible reasons for variation in adjustment to appearance change could be due to individual differences in appearance-related cognitions, such as levels of appearance investment or perceived visibility of the condition. As highlighted in recent research concerning other appearance-altering pediatric conditions (e.g. skin and craniofacial conditions), there is limited evidence concerning factors or processes which may exacerbate or ameliorate appearance (dis)satisfaction among these pediatric populations (Gee et al., 2020). Given the importance of body image development in adolescence, appearance satisfaction is recognized as a key outcome in AIS care (Negrini et al., 2006) and future research could seek to identify factors which may buffer against negative body image in AIS.

While participants acknowledged a changing appearance, their condition could be kept private and some were reluctant to explain their AIS to others. Explaining health conditions to peers has previously been identified as a challenge for adolescents receiving treatment within rheumatology, cardiology, and cystic fibrosis services (Secor-Turner et al., 2011; Kaushansky et al., 2017). As scoliosis can be concealed or of limited visibility, the decision to disclose is often left to the adolescent who may weigh up the threat to their desire for peer conformity and possible benefits such as social support from peers. Addressing adolescents’ health-related communication needs through educational strategies is therefore worth exploring in the context of AIS. Group-based psychoeducational programs aimed at promoting psychosocial wellbeing among adolescents with various chronic conditions have previously incorporated strategies to enhance communication (Last et al., 2007; Douma et al., 2019). Online and in-person learning activities focused on how and what adolescents may want to tell others about their health condition and taking initiative in informing others (e.g. at school, in peer groups) about issues related to their condition (e.g. reasons for absences, limitations).

The emotional burden of living with a serious health condition during adolescence is well recognized (Sawyer et al., 2007) and this was evident among participants as they came to terms with their AIS diagnosis and juggled fear and optimism in advance of surgery. Encouragingly, adolescents were able to gain emotional and practical support through communicating with others affected by AIS. They valued being able to share their experiences and were reassured by seeing others who had recovered from surgery. These findings build on those of MacCulloch et al. (2009) who reported that adolescents requested that their online scoliosis resource contain an interactive component to share personal stories. A peer support strategy may be of benefit to adolescents at the stage of surgical consideration and future research may seek to explore this further. Peer support for health conditions is understood to benefit participants by providing opportunity to learn coping techniques, acknowledge shared experiences, access encouragement and guidance, and reduce feelings of isolation (Olsson et al., 2005). Importantly, previous exploration of orthopedic healthcare providers recommendations for peer support highlights that such strategies should be professionally moderated (MacCulloch et al., 2010).

Surgical concerns voiced by the adolescents reiterate those reported in previous research related to surgery for AIS, including pain, surgical complications, and postsurgical limitations during the recovery period (Chan et al., 2017). However, the results of this qualitative investigation go beyond patients’ concerns to shed light on their positive expectations of surgery. Adolescents looked forward to regaining normality in their lives, returning to sports they enjoyed, and anticipated improvements in the appearance of their scoliosis. Focusing on positive expectations could be interpreted as a way of coping in advance of surgery for some of the adolescents. Notably, a previous study found that vigilant coping prior to surgery for AIS (defined as seeking information about surgery and recovery, acknowledging complications, and focusing on surgery benefits) was associated with higher levels of return to participation in recreational and social activities among adolescents in the 9 months after surgery (LaMontagne et al., 2004). Remaining cognizant of positive outcomes associated with AIS surgery may therefore be beneficial as part of preoperative preparation.

Although the optimism demonstrated by participants enabled them to view their upcoming surgery in a more positive light, it is also important to also consider management of realistic postoperative expectations. In particular, education about postsurgical appearance expectations is important for future satisfaction with surgical outcomes, as previous research found that higher presurgical expectations about postoperative appearance were associated with lower satisfaction 2 years after surgery (Sieberg et al., 2018). Spinal fusion surgery for AIS aims to prevent future progression and correct the existing curvature and while modern surgical techniques typically achieve curve correction of more than 60–70%, complete correction cannot be guaranteed (Imrie et al., 2011; Beauchamp et al., 2019).

### Limitations and Future Directions

As is typically the case with qualitative approaches, the study sample was limited to a relatively small cohort of patients. Recommendations regarding clinical implications can be considered as tentative suggestions based on the experience of this sample, which require further research and evaluation of their suitability or efficacy. The homogeneity of our sample means that findings are most relevant to adolescents with moderate-to-severe AIS at the stage of surgical consideration or presurgical preparation, rather than all patients with AIS at various stages of treatment. Furthermore, participants all identified as White Irish and were predominantly female. The gender breakdown is consistent with the higher incidence of AIS among females (Konieczny et al., 2013), however lack of ethnic or racial diversity within the sample may fail to capture experiences reflective of the broader population of patients with AIS. Further research exploring experiences of those with AIS at different stages of treatment and among diverse samples is warranted. Another factor to consider in the interpretation of findings is that anticipated time to surgery may have impacted some participant perspectives surrounding their treatment. However, it was not possible to estimate surgical wait times nor did we collect information on expected waiting times among this sample.

Building on the findings of this research, additional studies are required to qualitatively investigate the needs, goals, and the support preferences of presurgical adolescents with AIS, so that appropriate psychosocial interventions and supports can be developed. For example, preferred formats for strategies such as peer support could be considered (e.g. web-based, in-person). Future investigations should also seek to identify specific factors which may protect or buffer against negative psychosocial outcomes in AIS such as low body image, so that supports or interventions can be tailored accordingly. Additionally, given the role of parents in their child’s AIS care (Motyer et al., 2021), exploration of parental experiences throughout the presurgical period represents another avenue for future research to inform patient- and family-centered care in this area.

## Conclusion

This study has provided valuable insight into the experiences of adolescents with idiopathic scoliosis, developing an understanding of the ways in which adolescents negotiate psychological and social aspects of their condition throughout the presurgical stage of treatment. Findings have led to the consideration of strategies which may benefit the psychosocial wellbeing and functioning of presurgical patients with AIS. Strategies include guidance and promotion of suitable activity participation at various stages of treatment, addressing health-related communication needs, the development of and providing access to peer support networks, and preoperative assessment and counseling focused on positive postsurgical expectation management to promote effective coping and surgical outcomes. These practical implications can be applied both clinically and by those working with adolescents with AIS in community support settings. As conservative and operative treatment for AIS continue to advance in technique and efficacy, a concurrent focus on addressing psychosocial wellbeing and support needs is key in optimizing care and promoting best outcomes for this patient group.
